# Delayed frozen embryo transfer failed to improve live birth rate and neonatal outcomes in patients requiring whole embryo freezing

**DOI:** 10.1186/s12958-019-0560-1

**Published:** 2020-01-10

**Authors:** Yuxia He, Haiyan Zheng, Hongzi Du, Jianqiao Liu, Lei Li, Haiying Liu, Mingzhu Cao, Shiping Chen

**Affiliations:** 1 Department of Reproductive Medicine, the Third Affiliated Hospital of Guangzhou Medical University, 63 Duobao Road, Liwan District, Guangzhou, Guangdong China; 2 Key Laboratory of Reproductive Medicine of Guangdong Province, Guangzhou, Guangdong China; 3 Key Laboratory for Major Obstetric Diseases of Guangdong Province, Guangzhou, Guangdong China; 4 Key Laboratory of Reproduction and Genetics of Guangdong Higher Education Institutes, Guangzhou, Guangdong China

**Keywords:** Freeze-all strategy, Frozen embryo transfer, Endometrial receptivity, Live birth rate, Neonatal outcomes

## Abstract

**Background:**

Controlled ovarian stimulation (COS) has a negative effect on the endometrial receptivity compared with natural menstrual cycle. Whether it’s necessary to postpone the first frozen embryo transfer (FET) following a freeze-all strategy in order to avoid any residual effect on endometrial receptivity consequent to COS was inconclusive.

**Objective:**

The purpose of this retrospective study was to explore whether the delayed FET improve the live birth rate and neonatal outcomes stratified by COS protocols after a freeze-all strategy.

**Methods:**

A total of 4404 patients who underwent the first FET cycle were enrolled in this study between April 2014 to December 2017, and were divided into immediate (within the first menstrual cycle following withdrawal bleeding) or delayed FET (waiting for at least one menstrual cycle and the transferred embryos were cryopreserved for less than 6 months). Furthermore, each group was further divided into two subgroups according to COS protocols, and the pregnancy and neonatal outcomes were analyzed between the immediate and delayed FET following the same COS protocol.

**Results:**

When FET cycles following the same COS protocol, there was no significant difference regarding the rates of live birth, implantation, clinical pregnancy, multiple pregnancy, early miscarriage, premature birth and stillbirth between immediate and delayed FET groups. Similarly, no significant differences were found for the mean gestational age, the mean birth weight, and rates of low birth weight and very low birth weight between the immediate and delayed FET groups. The sex ratio (male/female) and the congenital anomalies rate also did not differ significantly between the two FET groups stratified by COS protocols.

**Conclusion:**

Regardless of COS protocols, FET could be performed immediately after a freeze-all strategy for delaying FET failed to improve reproductive and neonatal outcomes.

## Introduction

Many infertile couples benefits from assisted reproductive technologies (ART) including in vitro fertilization (IVF) or intracytoplasmic sperm injection (ICSI). Controlled ovarian stimulation (COS) is an important part of IVF, which can induce the development of multiple follicles and produce super-physiological levels of steroid hormones with the use of exogenous gonadotropin. Excessive stimulation of the ovaries can lead to life-threatening disease called ovarian hyperstimulation syndrome (OHSS). Then embryos have to be cryopreserved to avoid severe OHSS. Additionally, patients with extra-thin endometrium or increased progesterone level on the day of HCG administration [1, 2] will also fail to transfer fresh embryos. Therefore, frozen embryo transfer (FET) has progressively increased around the world since 1983 [3], and it has become one of the conventional clinical assisted reproductive techniques at present.

Successful embryo implantation depends on not only high quality embryos but also good endometrial receptivity whether for fresh embryo transfer or FET. Previous clinical studies showed that COS has a negative effect on the endometrial receptivity [4, 5]. Ovarian stimulation may be associated with poor endometrial vascularization, presented by decreased endometrial levels of natural killer cells and vascularization index in oocyte donors with stimulated cycles compared to natural cycles [6]. Other human studies also showed that endometrial receptivity was altered under COS compared with natural cycle from the same patients, indicated by disrupted transcriptional activation of genes involved in normal endometrial receptivity which affect endometrium gene expression profiles [5, 7]. Furthermore, there was evidence showed that impaired endometrial receptivity resulted from COS in fresh transfers have a detrimental effect on reproductive, maternal and perinatal outcomes compared to FET, and the negative effect of COS can be improved by using a freeze-all approach [8–10]. Regularly, patients will be given FET two menstrual cycles after COS, in order to avoid any residual effect on endometrial receptivity resulted from COS. But delayed FET may aggravate stress and anxiety to patients who has already been under pressure from infertility. And there’s no strong evidence to support the practice. Only a few studies were designed to explore different time interval between oocytes retrieval and a subsequent FET cycle on pregnancy outcomes, and the conclusions are indefinite and controversial [11–15].

As far as we know, there are no studies to evaluate the effect of performing FET during the first menstrual cycle following COS or delaying FET to subsequent cycles on reproductive and neonatal outcomes. To answer this unresolved question, this study was designed to compare the reproductive and neonatal outcomes of performing FET during the first menstrual cycle following COS versus delaying FET to subsequent cycles stratified by ovarian stimulation protocols.

## Materials and methods

### Study population and grouping

This was a retrospective cohort study conducted at the Department of Reproductive Medicine Center in the Third Affiliated Hospital of Guangzhou Medical University, Guangzhou, China. From April 2014 to December 2017, a total of 4404 patients were enrolled in this study. Inclusion criteria included: 1) women were 20 to 40 years of age, 2) had a normal menstrual cycle (defined as a spontaneous cycle length of ≥21 days and ≤ 35 days), 3) basal FSH<12mIU/ml, 4) the first FET cycle after whole embryo freezing using vitrification method, 5) COS regimens during IVF/ICSI cycle was the gonadotrophin releasing hormone agonist (GnRHa) or GnRH antagonist (GnRHant) protocol. Exclusion criteria included the following: 1) oocyte donation and cycles with preimplantation genetic testing (PGT), 2) patients were diagnosed with polycystic ovary syndrome (PCOS) or ovulation disorder, 3) known uterine anomalies confirmed by 3-dimensional sonography and/or hysteroscopy, including endometrial polyps, septal uterine cavity, submucucosal fibroid, etc.,4) the presence of hydrosalpinx not corrected surgically prior to FET, 5) uncontrolled endocrine and/or immune disorders or other systemic diseases, including hypertension, diabetes, thyroid disease, hyperprolactinemia, antiphospholipid syndrome, systemic lupus erythematosus, etc. Each patient has signed a informed consent on obtaining and analyzing their clinical data prior to the initiation of IVF/ICSI-ET treatment.

The FET cycles were divided in two groups according to the interval between withdrawal bleeding after ovum pick-up (OPU) and the start of the first FET. Immediate FET: women whose endometrial preparation of FET cycle performed within the first menstrual cycle following withdrawal bleeding; and delayed FET: women who had at least one menstrual cycle before initiation of endometrial preparation,and the transferred embryo were cryopreserved for less than 6 months. Furthermore, each group was divided into two subgroups according to COS protocols, and the reproductive and neonatal outcomes were analyzed between the immediate and delayed FET groups following the same COS protocol.

### Ovarian stimulation, vitrification,and warming

Patients in this study were given a long protocol treatment of down-regulation with GnRH agonist (Triptorelin; Diphereline, Ipsen, France) or GnRH antagonist (Cetrorelix; Cetrotide, Merck, Germany) protocol for ovarian stimulation. Individually tailored doses of recombinant human follitropin (Gonal F, Merck Serono, Switzerland or Puregon, MSD, Netherlands) were administered and then adjusted dosage based on the follicular development indicated by ultrasound monitoring and serum estradiol levels. Urine human chorionic gonadotrophin (uHCG; Lizhu Group Co., China) or recombinant HCG (r-HCG; Merck Serono) was administered to induce oocyte maturation when at least three follicles reached a mean diameter of 18 mm. Oocyte retrieval was performed by 36–38 h after HCG injection and oocytes were incubated in incubators for insemination by conventional IVF or ICSI detemined by sperm quality.

The cleavage stage embryos (day 3) or blastocysts (day 5/6) were graded and scored using the Garden criteria [16], and all available embryos were cryopreserved by vitrification method according to manufacturer’s instruction based on followed indications: high risk of ovarian hyperstimulation syndrome (OHSS), thin endometrium, increased progesterone level on the day of HCG administration and hydrosalpinx which then corrected surgically. Vitrified embryos were thawed by a rapid thawing method on the morning of embryo transfer. The number and stage of transferred embryos was determined by clinicians and the couples, giving priority to clinical factors including patients’ age, embryo qualities, number of available embryos.

### Endometrial preparation for FET cycle, and embryo transfer

Endometrial preparation for FET cycle in this study was achieved by natural cycle (NC) or hormone replace treatment (HRT) programs. The ovulation in NC program was determined by monitoring follicular development with transvaginal ultrasonography and hormone levels. The patients in HRT-FET cycles were treated with daily oral estradiol valerate tablets (Progynova, Bayer, Germany) since the second to fourth day of menstruation. When the endometrial thickness reached 7 mm or thicker, 40 mg/day progesterone was intramuscularly administered daily.

One or two thawed embryos were transfer on the fourth (cleavage-stage embryo) or sixth day (blastocyst) after ovulation or progesterone injection using a soft-tipped Wallace (PortexLed., Hythe, United Kingdom) catheter under ultrasound guidance. All patients received luteal support with progesterone after embryo transfer. If transvaginal ultrasound showed gestational sac and embryonic heartbeat 4–6 weeks after embryo transfer, luteal support was continued until 10 weeks of gestational age.

### Outcome measure

The primary reproductive outcome of this study was the live birth rate (LBR). Secondary endpoints included implantation, clinical pregnancy and spontaneous miscarriage rates. Neonatal outcomes included preterm birth, stillbirth, birth weight, low birth weight and congenital anomalies. Live birth was defined as the delivery of any viable neonate who was 28 weeks of gestation or older, and twins delivered by one mother were calculated as one live birth. Clinical pregnancy was defined as the present of gestational sac on ultrasound at 6–8 weeks of gestation; low birth weight was defined as the birth weight less than 2500 g and very low birth weight less than 1500 g.

### Statistical analysis

The statistical analysis was performed using the Statistical Package for Social Science (SPSS) version 22.0. The baseline characteristic was expressed as the mean ± SD (standard deviation) and differences in variables were compared by means of Student’s t-test. Categorical variables were described as frequencies and percentages, and compared using chi-square test and Fisher’s exact test when the number of events was less than 5. *P* < 0.05 was considered statistically significant.

## Results

A total of 4404 first FET cycles after a freeze-all strategy met study inclusion criteria and were included into the analysis. All FET cycles were divided into four groups according to the COS protocols and the intervals between the withdrawal bleeding after OPU and the initiation of endometial preparation for FET. There were 1585 cycles included in the immediate FET-GnRHa group, 1525 cycles in the delayed FET-GnRHa group, 778 cycles in the immediate FET-GnRHant group and 518 cycles in the delayed FET-GnRHant group. A total of 31 pregnant patients were lost to follow-up in this study.

Patients’ demographic and cycle characteristics of FETs between the immediate and delayed FET groups stratified by COS protocols are shown in Table 1.When FET cycles following the same COS protocol, there were no differences between the immediate and delayed FET groups in patients’age, body mass index (BMI), AMH, basal hormonal profile, infertility duration, types and causes of infertility, and endometrial thickness. Similarly,the mean number of retrieved oocytes and transferred embryos, age of transferred embryos and type of FET cycle did not vary between the two FET groups.

**Table 1:** Demographic and cycle characteristics of patients between immediate and delayed groups stratified by COS protocol

	GnRHa protocol			GnRHant protocol		
	Immediate FET (*n* = 1585)	Delayed FET (*n* = 1525)	*P* value	Immediate FET (*n* = 778)	Delayed FET (*n* = 516)	*P* value
Age (years)	31.74 ± 4.33	31.98 ± 4.23	0.113	31.37 ± 4.03	31.53 ± 4.23	0.190
BMI (kg/m^2^)	21.51 ± 3.01	21.43 ± 2.83	0.407	21.49 ± 2.83	21.36 ± 2.81	0.227
Type of infertility			0.062			0.606
Primary infertility	46.6 (738/1585)	49.9 (761/1525)		55.5 (432/778)	54.1 (279/516)	
Secondary infertility	53.4 (847/1585)	50.1 (764/1525)		44.5 (346/778)	45.9 (237/516)	
Infertility duration (years)	4.38 ± 2.95	4.45 ± 3.03	0.857	4.20 ± 2.83	4.39 ± 2.89	0.236
AMH (ng/ml)	3.17 ± 1.51	3.20 ± 1.12	0.324	3.34 ± 1.50	3.29 ± 1.63	0.788
FSH (mIU/ml)	5.45 ± 1.71	5.32 ± 1.78	0.269	5.31 ± 1.65	5.43 ± 1.69	0.309
LH (mIU/ml)	3.75 ± 1.57	3.67 ± 1.48	0.859	3.82 ± 1.58	3.77 ± 1.69	0.808
E_2_ (pmol/ml)	132.01 ± 48.75	135.71 ± 48.26	0.660	134.19 ± 49.67	131.77 ± 58.33	0.970
Reasons of infertility			0.424			0.321
Female factor	50.3 (798/1585)	52.9 (806/1525)		47.6 (370/778)	51.7 (267/516)	
Male factor	19.2 (305/1585)	19.3 (295/1525)		13.1 (102/778)	13.8 (71/516)	
Multiple factors	28.0 (444/1585)	25.7 (392/1525)		38.6 (300/778)	33.5 (173/516)	
Unexplained infertility	2.4 (38/1585)	2.1 (32/1525)		0.8 (6/778)	1.0 (5/516)	
No. of oocytes retrieved	15.81 ± 5.36	15.89 ± 5.43	0.709	15.63 ± 5.96	15.10 ± 6.40	0.328
Fertilization method			0.248			0.304
IVF	84.3 (1336/1585)	82.8 (1262/1585)		83.4 (649/778)	81.2 (419/778)	
ICSI	15.7 (249/1585)	17.2 (263/1585)		16.6 (129/778)	18.8 (97/778)	
Endometrial thickness on day of FET decision (mm)	9.14 ± 1.62	8.93 ± 1.65	0.001	8.79 ± 1.57	8.68 ± 1.67	0.234
No. of embryos transferred	1.80 ± 0.40	1.81 ± 0.40	0.713	1.68 ± 0.47	1.69 ± 0.46	0.652
Embryo developmental stage at transfer			0.463			0.064
Day 3 embryo	26.3 (417/1585)	28.5 (435/1525)		23.4 (182/778)	28.1 (145/516)	
Day 4embryo	3.5 (56/1585)	3.8 (58/1525)		3.3 (26/778)	3.1 (16/516)	
Day 5embryo	57.0 (903/1585)	54.3 (828/1525)		60.5 (471/778)	60.1 (310/516)	
Day 6 embryo	13.2 (209/1585)	13.4 (204/1525)		12.7 (99/778)	8.7 (45/516)	
Endometrial preparation			0.131			0.271
Natural	13.8 (218/1585)	15.7 (239/1525)		17.4 (135/778)	19.8 (102/516)	
Programmed	86.2 (1367/1585)	84.3 (1286/1525)		82.6 (643/778)	80.2 (414/516)	
Survival rate	97.9 (3136/3203)	97.7 (3237/3313)	0.520	98.2 (1448/1474)	98.5 (972/987)	0.643

Comparisons of FET pregnancy outcomes between the immediate and delayed FET groups stratified by COS protocols were summarized in Table 2. When FET cycles following the same COS protocol, there were no significant differences regarding the rates of implantation, clinical pregnancy, multiple pregnancy, spontaneous miscarriage and ectopic pregnancy between immediate FET and delayed FET groups. Meanwhile, the rates of live birth, premature birth and stillbirth (Table 3) were similar among immediate FET and delayed FET groups sub-grouped by COS protocols.

**Table 2:** Pregnancy outcomes of FETs between immediate and delayed groups stratified by COS protocol

	GnRHa protocol			GnRHant protocol		
	Immediate FET (*n* = 1585)	Delayed FET (*n* = 1525)	*P* value	Immediate FET (*n* = 778)	Delayed FET (*n* = 516)	*P* value
Implantation rate	45.7 (1245/2727)	45.1 (1241/2753)	0.668	45.7 (1245/2727)	45.1 (1241/2753)	0.668
Clinical Pregnancy rate	60.4 (957/1585)	59.7 (911/1525)	0.715	62.7 (488/778)	61.8 (319/516)	0.743
Multiple pregnancy rate	31.7 (303/957)	35.1 (320/911)	0.112	31.8 (155/488)	36.4 (116/319)	0.176
Monozygotic twins rate	2.4 (23/957)	2.5 (23/911)	0.866	2.0 (10/488)	1.9 (6/319)	0.867
Early spontaneous miscarriage rate	14.8 (142/957)	13.0 (118/911)	0.239	12.1 (59/488)	12.9 (41/319)	0.748
Spontaneous miscarriage rate	16.6 (159/957)	14.7 (134/911)	0.258	15.8 (77/488)	15.4 (49/319)	0.873
Ectopic pregnancy rate	1.6 (15/957)	1.5 (14/911)	0.957	1.6 (8/488)	2.2 (7/319)	0.568
Live birth rate	48.6 (771/1585)	48.8 (744/1525)	0.936	51.2 (398/778)	51.0 (263/516)	0.947

**Table 3:** Neonatal outcomes of FETs between immediate and delayed groups stratified by COS protocol

	GnRHa protocol			GnRHant protocol		
	Immediate FET (*n* = 1585)	Delayed FET (*n* = 1525)	*P* value	Immediate FET (*n* = 778)	Delayed FET (*n* = 516)	*P* value
Preterm birth (<37 weeks)	24.4 (189/774)	26.4 (197/745)	0.365	23.1 (92/399)	19.4 (51/263)	0.262
Still birth	0.4 (3/774)	0.1 (1/745)	0.625	0.3 (1/399)	0 (0/263)	1.000
Gestational age (weeks)	37.41 ± 2.44	37.46 ± 2.29	0.731	37.45 ± 2.45	37.57 ± 2.27	0.515
Birth height (mm)	49.04 ± 2.68	48.91 ± 2.36	0.356	48.37 ± 3.27	48.58 ± 2.84	0.179
Birth weight (kg)	3.00 ± 0.62	2.99 ± 1.15	0.891	2.93 ± 0.64	2.91 ± 0.60	0.548
Low birth weight (<2500 g)	22.8 (225/988)	25.1 (247/983)	0.221	21.4 (106/496)	19.7 (66/335)	0.560
Very low birth weight (<1500 g)	1.6 (16/988)	1.8 (18/983)	0.718	2.2 (11/496)	2.4 (8/335)	0.872
Congenital anomalies	1.0 (10/988)	0.3 (3/983)	0.053	2.2 (11/496)	1.2 (4/335)	0.277
Sex ratio (male/female)	1.17 (532/456)	1.17 (529/454)	0.989	1.28 (278/218)	1.31 (190/145)	0.849

Comparisons of neonatal outcomes between the immediate and delayed FET groups stratified by COS protocols were presented in Table 3.When FET cycles following the same COS protocol, no significant differences were found for the mean gestational age, the mean birth weight, and rates of low birth weight and very low birth weight between the immediate and delayed FET groups. The sex ratios (male/female) and incidences congenital anomalies did not differ significantly between the two FET groups stratified by COS protocol. Twenty-eight infants presented defects in the circulatory system (patent ductus arteriosusor ventricular septum defect), nervous system,digestive system (congenital esophageal atresia), circulation system (hemangioma), musculoskeletal system, external ear, cleft lip and palate.

## Discussion

Embryo cryopreservation has become an important part of ART around the world and plays a crucial role in those patients who are not suitable for fresh embryo transfer and require whole embryo freezing due to various situations [2, 3]. Following the vitrified-warmed cycles after a freeze-all strategy, the optimal time interval between withdrawal bleeding after OPU and subsequent FET cycle has been controversial. Here, our study showed that there were no significant differences in reproductive and neonatal outcomes between immediate and delayed FET cycles, suggesting the impaired endometrial receptivity due to COS might recover after the first withdrawal bleeding following OPU.

COS has a negative effect on endometrial receptivity, which is reflected in the changes of endometrial morphology and molecular expression during endometrial implantation period. The expression of pinopode and NCS (nucleolar channel systems) is an important morphological indicator for evaluating endometrial receptivity [17, 18]. Previous studies have showed that endometriumin IVF cycles depicted premature expression of pinopodes and NCS when compared with natural cycles [19–21]. These changes are associated with advanced endometrial maturation, which can lead to dyssynchrony between the uterine lining and the embryo in COS cycles [20]. Studies exploring the effects of COS on endometrial gene expression found that altered expression of some immunohistochemical marker of endometrial receptivity in COS cycle during midluteal endometrium when compared with natural cycle, including integrin, cytokine, chemokineand growth factor,which may suggest the detrimental effect of COS on endometrial receptivity [22–24]. A randomized controlled trial has shown higher pregnancy rates and better perinatal outcomes with frozen embryo transfer than with fresh embryo transfer [25], providing evidence of impaired endometrial receptivity during COS cycles.

As for the time patients wait before FET has not reached consensus to avoid the ‘potential’ residual adverse effect of COS on endometrial receptivity. One retrospective study including more than 1000 cycles clearly showed similar clinical pregnancy rates between immediate (32.5%)and delayed FET (31.7%) following failed fresh embryo transfers [13]. Another retrospective study with 333 FET cycles from the same research group suggested that the first FET performed immediately for women who underwent a freeze-all approach did not vary significantly from delayed FET in terms of clinical pregnancy rates [11]. However, those studies included only FET cycles following ovarian stimulation with GnRH antagonist cycles, and this conclusion should not be assumed as valid surrogates for the potential carryover effect of COS on pregnancy outcomes following GnRH agonist protocol. To this extent, another research team found significantly higher live birth rate in the non-adjacent group than in the adjacent group (32.3% versus 13.4%,*P* = 0.01), and support the postponement of FET after a failed fresh transfer when a preceding long GnRH-agonist protocol was used [12]. The literatures mentioned above on the optimal FET timing were paradoxical due to the different COS protocol.

Two previous studies, including GnRH-a or GnRH-ant protocols, have evaluated to the possible effect of timing of FET on reproductive outcomes. Kaye et al. found that there may be a clinically significant, though not statistically significant advantage of delayed FETand suggested a potential benefit in delaying a menstrual cycle before proceeding with FET [15]. Lattes et al. showed that there was no difference in live birth rate, among 512 FET cycles after a freeze-all strategy, between immediate and delayed FET after adjusting for numerous confounders (odds ratio, OR 0.73; 95% CI 0.49–1.08, 14]. However, those studies did not explore the effect of the FET timing on reproductive outcomes stratified by COS protocols. Moreover, none of them analyze the potential effect on other neonatal outcomes, such as preterm birth, birth weight, and so on.

Our study is the first one to investigate the reproductive and neonatal outcomes of performing FET during the first menstrual cycle following COS versus delayed FET to subsequent cycles stratified by COS protocols. Our results are consistent with previous viewpoints that following the same COS protocol, there was no significant difference in the rates of implantation, clinical pregnancy, miscarriage and live birth between immediate FET and delayed FET groups [13, 14]. This study confirmed that performing FET immediately after the first withdrawal bleeding following OPU did not affect the live birth rate regardless of the COS protocols. Additionally, our study showed that there were no significant differences in preterm birth, gestational age, birth weight, congenital anomalies and sex ratio between immediate FET and delayed FET groups stratified by COS protocols, which indicated COS did not have a carryover effect on neonatal outcomes and patients can prepare for FET cycle without delay.

The primary limitation of our study is the retrospective nature. However, as far as we know, the number of patients included in this study among each group was larger than other similar research, so that the results from this retrospective study are valuable for guiding the clinical practice to encourage physicians to schedule FET without hesitation. Meanwhile, this study analyzed the effect of FET timing on neonatal outcomes, and the results from our larger sample offer a more accurate inference for women wishing to become pregnant as soon as possible.

## Conclusions

This is, to our knowledge, the first study to explore the effect of postponing FET on live birth rate and neonatal outcomes, which showed that delayed FET failed to improve live birth rate and neonatal outcomes in patients requiring whole embryo freezing regardless of GnRH-a or GnRH-ant COS protocols. The finding of the current study suggested there is no benefit to postpone FET for additional menstrual cycles, and that could relive patient’s emotional stress and frustration associated with infertility. These results still need to be confirmed by prospective, randomized and controlled studies.

## Data Availability

The data sets used and/or analyzed during the current study are available from the corresponding author on reasonable request.
